# General Counteraction Exerted by Sugars against Denaturants

**DOI:** 10.3390/life11070652

**Published:** 2021-07-04

**Authors:** Serena Cozzolino, Attila Tortorella, Pompea Del Vecchio, Giuseppe Graziano

**Affiliations:** 1 Dipartimento di Scienze Chimiche, Università degli Studi di Napoli Federico II, Complesso Universitario di Monte Sant’Angelo, Via Cintia, 80126 Napoli, Italy; cozzolino@ill.fr (S.C.); at.tortorella@studenti.unina.it (A.T.); pompea.delvecchio@unina.it (P.D.V.); 2 Dipartimento di Scienze e Tecnologie, Università degli Studi del Sannio, Via Francesco de Sanctis snc, 82100 Benevento, Italy

**Keywords:** globular proteins, conformational stability, sugars, denaturants, solvent-excluded volume effect, solution density

## Abstract

The conformational stability of globular proteins is strongly influenced by the addition to water of different co-solutes. Some of the latter destabilize the native state, while others stabilize it. It is emerging that stabilizing agents are able to counteract the action of destabilizing agents. We have already provided experimental evidence that this counteraction is a general phenomenon and offered a rationalization. In the present work, we show that four different sugars, namely fructose, glucose, sucrose, and trehalose, counteract the effect of urea, tetramethylurea, sodium perchlorate, guanidinium chloride, and guanidinium thiocyanate despite the chemical and structural differences of those destabilizing agents. The rationalization we provide is as follows: (a) the solvent-excluded volume effect, a purely entropic effect, stabilizes the native state, whose solvent-accessible surface area is smaller than the one of denatured conformations; (b) the magnitude of the solvent-excluded volume effect increases markedly in ternary solutions because the experimental density of such solutions is larger than that of pure water.

## 1. Introduction

Protein stability and activity are influenced by several factors, like pressure [1,2,3,4], temperature [1,4,5,6], and presence of co-solutes in the studied system [6,7,8,9,10,11,12,13,14,15,16,17]. These co-solutes can both stabilize [10,12,14,18,19,20,21,22] or destabilize [15,16,17,23,24,25,26] the folded structure of a protein depending on their nature and the type and strength of interactions they make with the protein groups and/or the solvent molecules. Denaturing agents are described as molecules that preferentially interact with the polypeptide chain and are thus capable of shielding it from the solvent [15,16,17,27,28,29], while the effect of stabilizing agents is explained invoking a crowding action, i.e., they hinder part of the space in solution, forcing the protein molecule towards the more compact, folded conformation [10,12,14,27,28]. 

A huge literature exists on this topic, but most papers deal with a specific class of co-solutes (e.g., sugars [9,22,30,31,32], salts [27,33,34], amino acids [18,35]) instead of aiming at providing a general explanation (which has been, nevertheless, endeavoured in some cases [7,8,13,36]). 

Various co-solutes are key elements to understand issues in a number of fields, from biology and medicine [37,38] to astrobiology and studies on the origin of life [3,6]. For example, trimethylamine-N-oxide, TMAO, is found in vivo in fish, which produce it to counteract the denaturing action of urea, produced in vivo as well [39], or the destabilizing effect of high pressure in deep sea [6]; on the other hand, its precursor trimethylamine, TMA, is involved in cardiovascular and muscle cell disfunctions [37,38]. The denaturant perchlorate ion instead has been shown to shift the position of peak activity of α-chymotrypsin, allowing it to work at higher pressures [3]; this last example demonstrates that a greater conformational stability does not mean better functionality, i.e., a delicate balance exists in vivo among the various elements of a system, which sometimes gives unexpected results. This has to be borne in mind when using co-solutes to mimic the cellular environment for in vitro studies because it has been shown that the crowded environment in a cell is not accompanied by an exceptional conformational stability of the globular proteins in the system [40,41,42,43].

The knowledge of the behaviour of different co-solutes and a proper description of their effect is then of broad relevance. It is also interesting to examine the combined action of stabilizing and destabilizing agents, which has been mostly dealt with only in specific cases like the already cited TMAO/urea instance [16,20,22,24,27,33,34,44,45], with a few examples of comprehensive studies [7,8,33,36].

We already attempted [36] to give a general description of stabilizing and destabilizing agents using calorimetric measurements combined with a theoretical approach based on classic scaled particle theory (SPT) [46]. To apply this model, we must first recognise that each solute, when dissolving in a solvent, needs a void space (i.e., a cavity) to be accommodated in the solvent or solution. This leads to the solvent-excluded volume effect [23,25,32,47,48], which poses a geometric constraint on the molecular location and translational motion in a solution: solute molecules occupy a certain space, and their presence means that this occupied space is no longer available to host solvent molecules. The magnitude of this effect can be determined by means of suitable statistical mechanical models and/or computer simulations, using the experimentally measured density of the solution from which the number density and the volume packing density of the solution itself, two key parameters of the theoretical model, can be derived.

Here, we apply again differential scanning calorimetry (DSC) to study the conformational stability of RNase A in the presence of various co-solutes, but we focus on some sugars, namely fructose, glucose, sucrose, and trehalose, and show how they are all able to counteract the effect of the denaturants urea, tetramethylurea (TMU), sodium perchlorate (NaClO_4_), guanidinium chloride (GdmCl), and guanidinium thiocyanate (GdmSCN). The devised theoretical approach will try to explain the common origin of the general counteraction observed in the experimental results.

## 2. Materials and Methods

### 2.1. Materials

Pancreatic ribonuclease, RNase A (Type XII-A), urea, TMU, GdmCl, fructose, glucose, sucrose, and trehalose were from Sigma; GdmSCN was from Fluka; and sodium perchlorate was from Carlo Erba. A buffer solution of 10 mM MOPS (3-(N-morpholino) propanesulfonic acid) with 100 mM NaCl at pH 7.0 was used. MOPS buffer was chosen for its low protonation enthalpy to reduce the pH temperature dependence. Chemicals for buffer were of analytical grade from Sigma and dissolved in Milli Q (Millipore, Bedford, MA, USA) water. Urea stock solutions were prepared by weight in a 3 mL final volume calibrated flask. Urea solutions were prepared just before use. GdmCl and GdmSCN were purchased as a ready-to-use 8 M and 6 M buffered solutions, respectively. Stock solutions of sugars were prepared by weight in a 10 mL final volume calibrated flask. Protein stock solutions were extensively dialyzed against the buffer and their concentration determined by UV spectra using a sequence-based extinction coefficient of 9440 M^−1^ cm^−1^ at 280 nm. Samples for DSC measurements were prepared by mixing appropriate volumes of the protein stock solution with co-solute stock solutions, then diluting with buffer up to a fixed volume of 2 mL in a calibrated flask. Protein final concentration was kept constant at 1 mg mL^−1^.

### 2.2. DSC Measurements

DSC measurements were conducted on a Nano-DSC (TA Instruments, USA), which has an active cell volume of 0.3 mL and works at a pressure of 3 atm. The scan speed was set at 1 °C min^−1^. Samples were prepared in a 3 mL calibrated flask by adding the appropriate amount of protein and co-solute(s) and diluting with buffer until reaching the final volume. Each measurement was done in triplicate, measuring the appropriate blank (buffer or buffer plus co-solute(s)) before the sample, and two scans were performed on each sample to check the reversibility of the thermal denaturation. Data were analysed using the Nano-Analyze software supplied by the manufacturer: the blank scan was subtracted by the sample scan and the differential heat capacity, <ΔC_p_>, was obtained by taking as baseline the linear temperature dependence of the heat capacity of the protein native state [49]. The calorimetric enthalpy change, ΔH_d_, was then obtained by integrating the area under the curve. Using the Nano-Analyze software, the experimental DSC curve was modelled with a theoretical two-state curve to calculate the Van ’t Hoff enthalpy change, ΔH_d_^vH^, and from this value, the cooperative unit, CU, defined as the calorimetric to Van ’t Hoff enthalpy ratio. A CU value near 1 indicates that the observed transition is a two-state process, a necessary condition for the performed thermodynamic analysis [50]. The heat capacity change of denaturation, ΔC_p,d_, was obtained from the slope of a ΔH_d_ *versus* T_d_ plot, as already described [36,44]. The ΔC_p,d_, ΔH_d_ and T_d_ values were then employed to calculate for each system the Gibbs energy of denaturation at 25 °C, ΔG_d_(25 °C), via the Gibbs–Helmholtz equation [15].

### 2.3. Density Measurements

The density of binary (containing water and a sugar or a denaturant) and ternary (containing water and both a sugar and a denaturant) solutions was measured using a vibrating tube densimeter (Anton Paar 5000, Austria), which has an accuracy of 0.5 g dm^−3^, at a constant cell temperature of 25.000 ± 0.001 °C. Samples were prepared and measured in triplicate. For the preparation, a 2 mL calibrated flask was employed; the required amount of co-solute(s) was taken by weight, and then water was added until reaching the final volume.

### 2.4. Theoretical Approach

It is important to remind that water plays a fundamental role for the conformational stability of globular proteins, but its action is not due to the formation of ordered structures akin to icebergs or clathrates around nonpolar moieties. The structural reorganization of water molecules upon insertion of a nonpolar solute is characterized by an almost complete enthalpy-entropy compensation and so cannot affect the overall Gibbs energy change [51,52,53]. Instead, the action of water is associated with cavity creation, that is, the theoretical means to account for the loss in translational entropy that water molecules have to pay in order to host the solute molecules (i.e., the entrance of a solute molecule in a liquid needs the presence of a void space to host it). The statistical mechanical approach indicates that the Gibbs energy change for the denaturation of a globular protein, ΔG_d_, can be divided into three contributions (Equation (1)) [23,25,48], two of which are entropic terms related to the translational freedom of solvent and co-solute molecules for cavity creation, and to the conformational freedom of the polypeptide chain (ΔΔG_c_ and T·ΔS_conf_, respectively), and the third one, ΔE_a_, is due to the variation of energetic attractions upon denaturation [23,25,32,36,44,48]:

ΔG_d_ = ΔΔG_c_ − T·ΔS_conf_ + ΔE_a_ (1)

ΔΔG_c_, defined in Equation (2), represents the difference in the Gibbs energy change for cavity creation between the denatured D-state and native N-state conformations of a protein, assumed as the only two populated macro-states of the system:

ΔΔG_c_ = ΔG_c_(D-state) − ΔG_c_(N-state) (2)

The ΔG_c_ terms are related to geometric parameters and to the density of the solution, as explained in previous works [23,25,32,36,44,47]. It is perhaps worth underscoring the importance of this term, recalling here its meaning. For the cavity to be created, we can define size and shape, which are, respectively, the van der Waals volume, V_vdW_, of the protein and its solvent-accessible surface area, SASA, i.e., a shell surrounding the protein defined by the surface of the closest points to the protein surface that solvent and co-solute molecules can reach. Beyond this limit, the cavity starts, where the solvent and co-solute molecules cannot enter. Cavity formation at constant temperature and pressure causes an increase in the volume of the solution; nevertheless, the space occupied by the protein remains inaccessible to the solvent and co-solute molecules, limiting their movement in the solution: this is the aforementioned solvent-excluded volume effect. Keeping fixed V_vdW_, ΔG_c_ increases almost linearly with SASA, as shown by classic SPT calculations and computer simulations [54,55,56]. ΔG_c_ is also related to the density of the solution through the number density and the volume packing density parameters that will be presented in the following equation. Classic SPT [46] produces an equation for ΔG_c_ (Equation (3)) on the assumption that the solvent and co-solute molecules are described as hard spheres (an approximation that anyway allows to reach qualitatively right results [25,32,36,44]), while the protein is modelled as a sphere in the N-state and as a prolate spherocylinder in the D-state:

(3) $$\Delta G_{c}=RT\cdot \left\{-ln\left(1-\xi_{3}\right)+\left(6\frac{\xi_{2}}{1-\xi_{3}}\right)a+\left(12\frac{\xi_{1}}{1-\xi_{3}}\right)a^{2}+\left[18\frac{\xi_{2}^{2}}{{\left(1-\xi_{3}\right)}^{2}}\right]a^{2}+\;\left[3\frac{\xi_{2}}{2\left(1-\xi_{3}\right)}\right]l+\left(6\frac{\xi_{1}}{1-\xi_{3}}\right)a\cdot l+\left[9\frac{\xi_{2}^{2}}{{\left(1-\xi_{3}\right)}^{2}}\right]a\cdot l\right\}$$

In this formula, the terms ξ_i_ = (π/6)⋅Σρ_j_·σ_j_^i^ depend on the number density of the solution, ρ_j_, expressed in molecules per Å^3^, and on the diameter, σ_j_, of the hard sphere associated with each molecule j, so ξ_3_ = (π/6)⋅Σρ_j_·σ_j_^3^ represents the volume packing density of the solution. a and l are the radius and the length of the prolate spherocylinder representing the D-state; when l = 0, the equation can be referred to a spherical cavity, and a indicates the radius of the sphere associated with the N-state. This model implies that only one structure is chosen as representative of the D-state among the huge ensemble of conformations that actually exist; the choice is, however, in line with theoretical calculations showing that the D-state of globular proteins resembles a prolate ellipsoid [57]. Moreover, the conformations in the N- and D-states are described as having the same V_vdW_ (being the volume change upon denaturation a negligible quantity at the working condition of P = 1 atm [58,59,60]) but a larger SASA for the D-state. Reliable estimates for these quantities in the case of a 138-residue model globular protein are: for the N-state, a = 15 Å, V_vdW_ = 14,137 Å^3^ and SASA = 3380 Å^2^; for the D-state, a = 6 Å, l = 117 Å, V_vdW_ = 14,137 Å^3^ and SASA = 6128 Å^2^ (the SASA values are calculated based on the water molecule radius of 1.4 Å [61,62]) [44,63]. Debenedetti and co-workers [64] modelled both the N- and D-states as spheres, but this idea cannot be considered reliable. In fact, the sphere modelling the D-state has a larger radius than the one modelling the N-state, and so the two objects have very different values of V_vdW_, in contrast with experimental findings [58,59,60].

Concerning the other diameters σ_j_, indicated in Table 1, as in previous works [36,44], those of the sugars are taken from van der Waals volume-increment tables [65], with the exception of trehalose [47]; diameter values for urea, TMU, and guanidinium ion are referred to a sphere having their same SASA [66]; the diameters of chloride and perchlorate ions are the Pauling-type ones reported by Marcus [67]; that of thiocyanate corresponds to the hard sphere diameter of carbon dioxide [68].

Coming back to the equation for ΔG_d_, the term T·ΔS_conf_ (Equation (4)) describes the conformational entropy gain of the polypeptide chain upon denaturation. As each residue contributes a similar amount ΔS_conf_(res) = 19.0 J K^−1^ molres^−1^ to the overall value [25,36,44,48,63,69], this term can be estimated knowing the number of amino acid residues, N_res_, in the polypeptide chain:

T·ΔS_conf_ = T·ΔS_conf_(res)·N_res_ (4)

This quantity does not depend on the presence of co-solutes in solution if the denaturation occurs between the same two macro-states involved in the unfolding process in the absence of co-solutes [36,44].

The last term in Equation (1), ΔE_a,_ is defined in Equation (5), andaccounts for the difference of both intermolecular and intramolecular energetic attractions experienced by the protein in the D-state and in the N-state:

ΔE_a_ = E_a_(D-state) − E_a_(N-state) + ΔE_a_(intra) (5)

A reliable estimation of this term is complicated by the lack of details in the definition of the concerned energetic contributions: both a way to correctly estimate the magnitude of the various interactions and a thorough description of all the conformations explored by the protein in the D-state would be required [11,36,63]. Qualitatively, anyway, we can infer that, being the number of possible attractions greater with a larger SASA, i.e., in the denatured state, ΔE_a_ is a negative quantity [23,25,36,44]. This holds in solutions of both stabilizing and destabilizing agents, but its magnitude changes. Denaturing agents are known to preferentially interact with the protein surface [28], while in the case of stabilizing agents, van der Waals forces seem to play a major role, thus making the magnitude of the ΔE_a_ contribution greater in the aqueous solutions containing denaturants [36,47]. It is great enough to overcome, in these systems, the ΔΔG_c_ contribution, which is instead a positive quantity acting in the opposite direction (it favours the more compact N-state) [11,23,25,36,44,48,63,69]. The consequences of the presence of co-solutes, then, are dictated by the balance between the ΔΔG_c_ and ΔE_a_ terms, whose relative magnitude determines whether the composition of an aqueous solution will stabilize or destabilize the native conformation of a protein [27,28,40,70].

It is important to underscore that the thermodynamic quantities appearing in the theoretical approach have a molecular origin and so have nothing to do with the denaturation enthalpy and entropy changes obtained by means of DSC measurements. The latter are macroscopic thermodynamic quantities that take into account also the contributions coming from the structural reorganization of solvent and co-solute molecules upon denaturation [63] (i.e., upon the conformational modification of the polypeptide chain). The experimental ΔG_d_ quantity does not depend upon the structural reorganization of solvent and co-solute molecules because the latter is characterized by an almost complete enthalpy-entropy compensation [51,52,53] and so could be directly compared with the calculated ΔG_d_ one. Actually, we preferred to provide calculated estimates only of the ΔΔG_c_ term in view of the large uncertainties associated with the calculation of the ΔE_a_ term, given the missing information on the denatured state ensemble.

## 3. Results and Discussion

**Figure 1:**
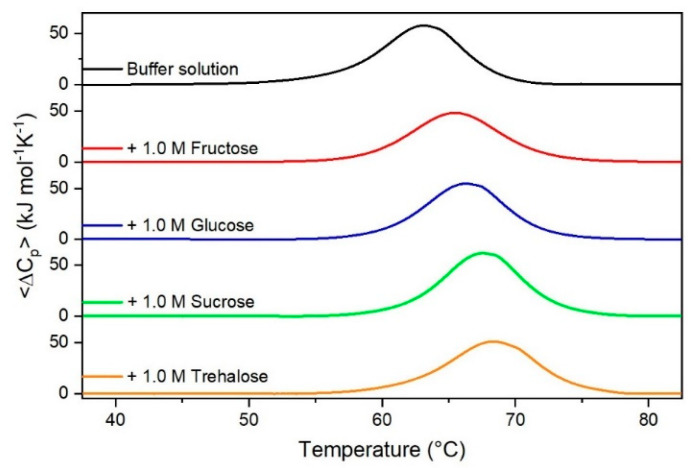
DSC curves of RNase A in the absence or in the presence of sugars as indicated in the Figure.

**Figure 2:**
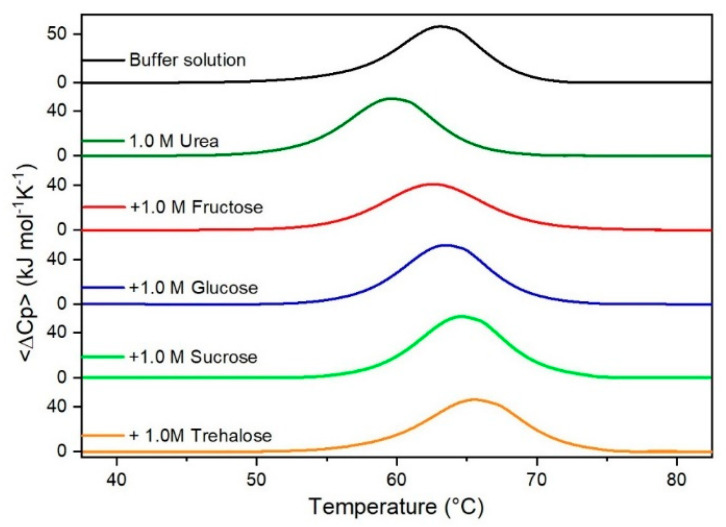
DSC curves of RNase A in 1.0 M urea in the absence or in the presence of the indicated sugars. For comparison, the DSC profile of the protein in the absence of co-solutes is reported (black curve).

**Figure 3:**
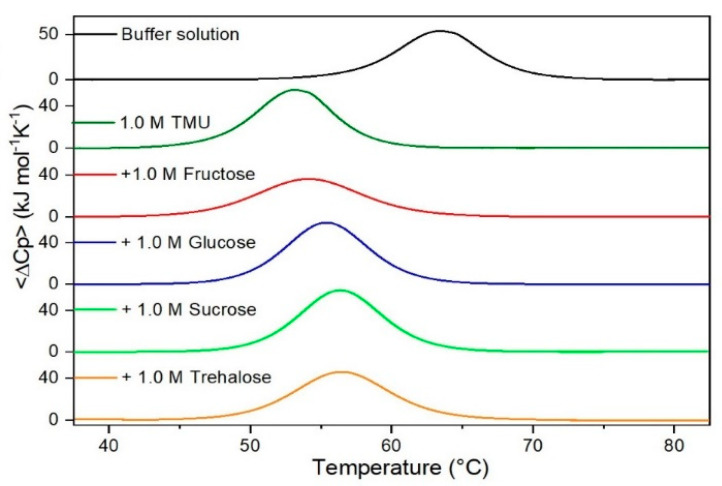
DSC curves of RNase A in 1.0 M TMU in the absence or in the presence of the indicated sugars. For comparison, the DSC profile of the protein in the absence of co-solutes is reported (black curve).

**Figure 4:**
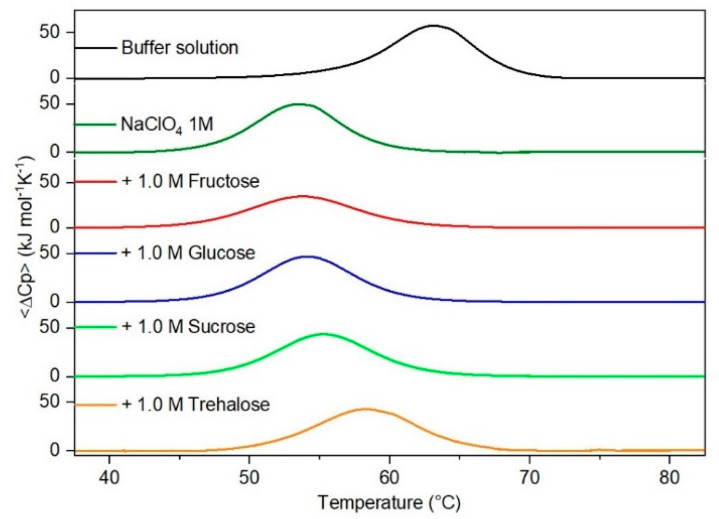
DSC curves of RNase A in 1.0 M NaClO_4_ in the absence or in the presence of the indicated sugars. For comparison, the DSC profile of the protein in the absence of co-solutes is reported (black curve).

**Figure 5:**
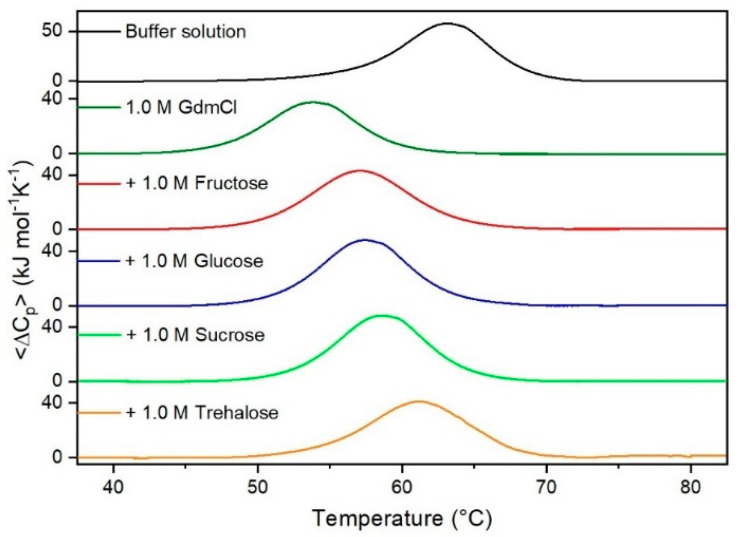
DSC curves of RNase A in 1.0 M GdmCl in the absence or in the presence of the indicated sugars. For comparison, the DSC profile of the protein in the absence of co-solutes is reported (black curve).

**Figure 6:**
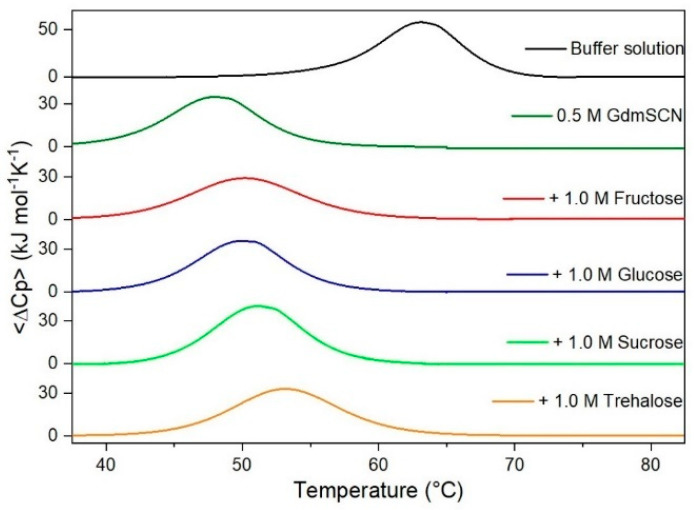
DSC curves of RNase A in 0.5 M GdmSCN in the absence or in the presence of the indicated sugars. For comparison, the DSC profile of the protein in the absence of co-solutes is reported (black curve).

It is important to underscore that the concentrations of both the stabilizing and the destabilizing co-solutes used in the present study (i.e., at most 1 M) do not modify the conformational features of the RNase A native state at 25 °C and pH 7.0, as indicated by far-UV CD measurements (data not shown). DSC measurements indicate that the temperature-induced unfolding of RNase A at pH 7.0 proves to be a reversible process in all the considered aqueous solutions (Figure 1, Figure 2, Figure 3, Figure 4, Figure 5 and Figure 6). The reversibility has been tested by means of the reheating criterion. The T_d_ values, listed in the third column of Table 2, prove that: (a) the protein thermal stability increases in the presence of sugars, whereas it decreases in the presence of urea, TMU, NaClO_4_, GdmCl, and GdmSCN; (b) GdmSCN is the most effective destabilizing agent (i.e., T_d_ = 47.9 °C at 0.5 M GdmSCN), and trehalose is the most effective stabilizing agent (i.e., T_d_ = 68.4 °C at 1 M trehalose); (c) the addition of these four sugars to the solutions containing the denaturants causes a counteraction of the destabilizing effect of the considered denaturants (i.e., it leads to a re-increase of the protein denaturation temperature); (d) fructose at 1 M concentration has a poor counterbalance activity against TMU and GdmSCN; (e) experimental conditions needed to achieve a complete counterbalance for each couple of stabilizing and destabilizing agents have not been searched. The counteraction ability is a property belonging to all the sugars and, since the denaturing agents have different chemical nature and structure, such an ability cannot originate from direct interactions between sugar molecules and denaturants; it must be grounded in some more basic features of the ternary aqueous solutions (i.e., those containing water and both a stabilizing agent and a destabilizing one).

On looking at the values of the denaturation enthalpy change, ΔH_d_(T_d_), listed in the fourth column of Table 2, it is evident that: (a) the denaturation process is well described as a reversible two-state N ⇄ D transition due to the closeness to one of the CU values (see the fifth column of Table 2); (b) all the four considered sugars do not affect significantly ΔH_d_(T_d_), suggesting an entropic stabilization mechanism that should be grounded in the solvent-excluded volume effect; (c) the addition of GdmCl or GdmSCN causes a marked decrease of the ΔH_d_(T_d_) values, suggesting the occurrence of preferential energetic attractions of the Gdm^+^ and SCN^−^ ions with the protein surface that destabilize enthalpically the native state favoring the denatured one, which possesses a larger SASA. It is well established that large molecular ions, such as Gdm^+^ and SCN^−^, characterized by a low charge density, do not have strong electrostatic interactions with water molecules and are more attracted by the nonpolar moieties on the protein surface [71,72]. Present DSC data do not support an enthalpic stabilization mechanism of sugars, in contrast to the findings of some authors [73,74]; what would be the molecular origin of the claimed enthalpic stabilization is absolutely not clear.

**Figure 7:**
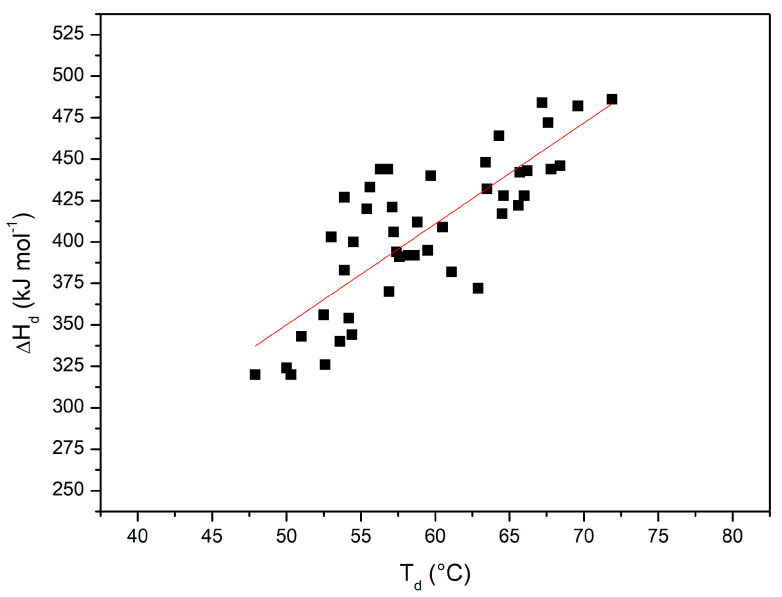
ΔH_d_(T_d_) versus T_d_ plot, with values from all the DSC measurements shown here and in [36].

Figure 7 shows the plot of ΔH_d_(T_d_) versus T_d_ with all the performed DSC measurements (i.e., considering also all those reported in [36]); the 45 points can be described by a straight line with a linear correlation coefficient *r* = 0.8 (note that the probability that 45 truly independent measurements of two uncorrelated variables produce a linear correlation coefficient equal to 0.8 is negligibly small; of course, the present DSC measurements are not truly independent of each other). The slope of this plot corresponds to the denaturation heat capacity change ΔC_p,d_ = 6.4 ± 0.7 kJ K^−1^mol^−1^. The latter number with the obtained uncertainty is in line with the ΔC_p,d_ values existing in the literature for RNase A [75], taking also into account that its magnitude can be affected by the presence of co-solutes (see the ΔC_p,d_ values in Table 4 of Makhatadze and Privalov [15]). The obtained average ΔC_p,d_ value has been used in the Gibbs–Helmholtz equation to estimate ΔG_d_ at 25 °C for RNase A in all the considered experimental conditions (see the last column of Table 2). The latter estimates, notwithstanding the large errors due to the needed long temperature extrapolation of direct DSC data, show a not so different rank order from that of T_d_ values.

An explanation of these results must be provided. The addition of both sugars and denaturants to water causes a density increase (see the second column of Table 1) that leads to an increase in the magnitude of the solvent-excluded volume effect (i.e., an increase of the stabilizing ΔΔG_c_ term). The density increase is a general effect due to the strength of the energetic attractions between the added co-solutes and water molecules. In fact, all of them, regardless of their stabilizing or destabilizing actions towards globular proteins, prove to be very soluble in water. Density plays the pivotal role because we are considering aqueous solutions in which water is by far the dominant component (see the third column of Table 1), and therefore, molecular dimensions play a minor role [48].

To make a step forward, it is necessary to consider energetic factors. The presence of a protein in solution implies the need to take into account the strength of co-solute—water energetic attractions that compare to the strength of both co-solute— protein and water—protein energetic attractions. In other words, an active trade-off is operative and controls the magnitude of the destabilizing ΔE_a_ term. It appears that sugars have stronger energetic attractions with water molecules than with protein groups, whereas denaturants have stronger energetic attractions with protein groups than with water molecules. In fact, sugar molecules, rich in OH groups, can saturate all their H-bonding capabilities interacting with water molecules [76] but can saturate only a fraction of their H-bonding capabilities interacting with polar groups on protein surfaces due to simple geometric-steric constraints. This molecular picture can rationalize the preferential exclusion mechanism advocated to explain the stabilizing action of sugars towards the N-state of globular proteins (note that the simple exclusion of sugar molecules from the solvation shell of proteins does not provide a clear stabilization of the N-state without taking into account the solvent-excluded volume effect). The energetic trade-off is delicate, and in general, very high concentrations of urea or GdmCl are required to cause the denaturation of a globular protein. The direct equilibrium constants of urea molecules or Gdm^+^ ions to protein groups are small, and the interaction cannot be described by a simple binding mechanism [15]. A more correct description should consider an exchange equilibrium between water and co-solute for an average protein site, as originally suggested by John Schellman [28]. It is important to underscore that computer simulations of coarse-grained models of both globular proteins and aqueous solutions (using effective potentials that account in an indirect manner for the presence and function of water and co-solute molecules) cannot provide a true understanding of the molecular mechanisms of stabilizing and destabilizing agents [77,78].

A reliable explanation of the molecular origin of counteraction ability is the following. In the case of ternary solutions, the density increase is large, and it is associated with an increase in the volume packing density, with an increase in the total number density, or with a combination of ξ_3_ rise and a small number density decrease with respect to pure water. All these situations imply that the ΔΔG_c_ term is very large and positive in ternary solutions and overwhelms the negative ΔE_a_ contribution (whose magnitude cannot increase indefinitely, as can be appreciated on looking at the results of computer simulations on model systems in aqueous 10 M urea [16] and aqueous 4.9 M GdmCl solutions [17]). In other words, even though urea molecules or Gdm^+^ and SCN^−^ ions have preferential energetic attractions with protein groups that lead to a large and negative ΔE_a_ contribution, the magnitude of the stabilizing ΔΔG_c_ term is larger and leads to an increase of protein thermal stability (i.e., the T_d_ value rises).

It is useful to write down some values. For the model small globular protein described in the Theoretical Approach section, ΔΔG_c_(in kJ mol^−1^ units) = 804 in water, 849 in 1 M GdmCl, 856 in 1 M glucose, 865 in 1 M sucrose, 916 in 1 M GdmCl + 1 M glucose, and 918 in 1 M GdmCl + 1 M sucrose (the situation is qualitatively similar in the other cases; see the values listed in the last two columns of Table 3). The significant ΔΔG_c_ increase characterizing the ternary solutions is the cause of counterbalance because it is able to overwhelm the energetic ΔE_a_ contribution coming from the preferential attractions of the denaturing agents with protein groups. Using different words, one could say that: (a) the preferential attractions of urea and TMU molecules or Gdm^+^ and SCN^−^ ions with protein groups are not hindered/destroyed by the presence of sugars in the aqueous solution; (b) however, the magnitude of the solvent-excluded volume effect increases so much that it stabilizes the N-state, whose SASA is markedly smaller than that of the D-state. This reasoning does not mean that the two co-solutes operate in an independent manner because the magnitude of the solvent-excluded volume effect depends upon the density, the volume packing density, and the number density of the solution, and the latter quantities are a manifestation of the geometric size and energetic interactions of all the species existing in solution. It is important to recognize that the solvent-excluded volume effect is non-additive by definition. A final point: it is worth underscoring that the stabilization mechanism of polysaccharides, like Dextran and Ficoll, even though grounded in the solvent-excluded volume effect, has to be considered more complex because the presence in solution of long and flexible polymeric chains produces different effects from those caused by small co-solutes [10,32,42].

In conclusion, DSC measurements on RNase A at neutral pH indicate that the considered four sugars are able to counteract the destabilizing action of urea, TMU, NaClO_4_, GdmCl, and GdmSCN. Those denaturants are chemically different and interact with the protein by means of various mechanisms; instead, the four sugars are chemically similar, but they show different structural features. Despite this, any stabilizer-destabilizer pair showed a counteraction of the denaturing effect seen in binary solutions of destabilizing agents. We already observed analogous effects using chemically diverse stabilizing agents [36,44]. So, notwithstanding the specificities that may be added for each system, our results point towards the existence of a common physical origin of the counteraction independent of the chemical and structural differences among the sugars and among the denaturants.

A reliable explanation could be: (a) the density of aqueous solutions is a fundamental factor because it determines the magnitude of the stabilizing solvent-excluded volume effect; (b) the addition of both sugars and denaturants causes a significant density increase due to their good, energetic interactions with water molecules; (c) this density increase is magnified in the case of ternary solutions, determining a general counteraction of the N-state destabilization operated by denaturants. We hope the present study will stimulate the realization of detailed MD investigations on the relevant aqueous ternary solutions to shed further light on the counteraction phenomenon.

## Figures and Tables

**Table 1:** Experimental values of the density and water molar concentration for pure water and for considered binary and ternary aqueous solutions at 25 °C and 1 atm; values of the effective hard sphere diameters, σ, assigned to all the species; values of the volume packing density, ξ_3_ for the solutions. Values in italics are taken from [36].

	ρ (g L^−1^)	[H2O] (M)	σ (Å)	ξ3
*H_2_O*	*997*	*55.3*	*2.80*	*0.383*
1.0 M Fructose	1066	49.2	6.40	0.423
*1.0 M Glucose*	*1062*	*48.9*	*6.60*	*0.429*
*1.0 M Sucrose*	*1126*	*43.5*	*8.10*	*0.469*
1.0 M Trehalose	1130	43.7	8.30	0.483
*1.0 M Urea*	*1013*	52.9	4.64	0.398
1.0 M Urea + 1.0 M Fructose	1082	46.7	4.64 and 6.40	0.437
*1.0 M Urea + 1.0 M Glucose*	*1080*	*46.6*	*4.64 and 6.60*	*0.445*
*1.0 M Urea + 1.0 M Sucrose*	*1143*	*41.1*	*4.64 and 8.10*	*0.484*
1.0 M Urea + 1.0 M Trehalose	1144	41.2	4.64 and 8.30	0.497
1.0 M TMU	999	49.0	6.13	0.412
1.0 M TMU + 1.0 M Fructose	1067	42.8	6.13 and 6.40	0.451
1.0 M TMU + 1.0 M Glucose	1068	42.8	6.13 and 6.60	0.460
1.0 M TMU + 1.0 M Sucrose	1127	37.1	6.13 and 8.10	0.497
1.0 M TMU + 1.0 M Trehalose	1131	37.3	6.13 and 8.30	0.511
*1.0 M NaClO_4_*	*1068*	52.5	2.02 and 4.80	0.401
1.0 M NaClO_4_ + 1.0 M Fructose	1144	46.7	2.02, 4.80, and 6.40	0.443
*1.0 M NaClO_4_ + 1.0 M Glucose*	*1136*	*46.3*	*2.02, 4.80, and 6.60*	*0.448*
*1.0 M NaClO_4_ + 1.0 M Sucrose*	*1198*	*40.7*	*2.02, 4.80, and 8.10*	*0.487*
1.0 M NaClO_4_ + 1.0 M Trehalose	1216	41.7	2.02, 4.80, and 8.30	0.506
*1.0 M GdmCl*	*1022*	*51.4*	*4.70 and 3.62*	*0.404*
1.0 M GdmCl + 1.0 M Fructose	1090	45.2	4.70, 3.62, and 6.40	0.443
*1.0 M GdmCl + 1.0 M Glucose*	*1091*	*45.2*	*4.70, 3.62, and 6.60*	*0.451*
*1.0 M GdmCl + 1.0 M Sucrose*	*1150*	*39.5*	*4.70, 3.62, and 8.10*	*0.489*
1.0 M GdmCl + 1.0 M Trehalose	1156	39.9	4.70, 3.62, and 8.30	0.504
*0.5 M GdmSCN*	*1011*	*52.8*	*4.70 and 3.94*	*0.392*
0.5 M GdmSCN + 1.0 M Fructose	1079	46.6	4.70, 3.94, and 6.40	0.431
*0.5 M GdmSCN + 1.0 M Glucose*	*1079*	*46.6*	*4.70, 3.94, and 6.60*	*0.439*
*0.5 M GdmSCN + 1.0 M Sucrose*	*1145*	*41.3*	*4.70, 3.94, and 8.10*	*0.479*
0.5 M GdmSCN + 1.0 M Trehalose	1141	41.0	4.70, 3.94, and 8.30	0.490

**Table 2:** Thermodynamic parameters for the thermal denaturation of RNase A in the absence and presence of the different sugars and denaturing agents, in 10 mM MOPS, 100 mM NaCl buffer solution, pH 7.0. Values in italics are taken from [36].

Co-Solute		^a^ T_d_ (°C)	^a^ ΔH_d_(T_d_) (kJ mol^−1^)	CU	ΔG_d_(25 °C) (kJ mol^−1^)
*-*	*-*	*63.4*	*448*	*1.0*	*36*
-	1.0 M Fructose	65.7	442	1.0	37
*-*	*1.0 M Glucose*	*66.2*	*443*	*0.96*	*37*
*-*	*1.0 M Sucrose*	*67.6*	*472*	*0.98*	*41*
-	1.0 M Trehalose	68.4	446	1.0	38
Urea					
*1.0 M*	*-*	*59.7*	*440*	*1.0*	*33*
1.0 M	1.0 M Fructose	62.9	372	0.95	28
*1.0 M*	*1.0 M Glucose*	*63.5*	*432*	*0.98*	*35*
*1.0 M*	*1.0 M Sucrose*	*64.6*	*428*	*0.96*	*35*
1.0 M	1.0 M Trehalose	65.6	422	0.99	34
TMU					
1.0 M	-	53.9	427	1.0	29
1.0 M	1.0 M Fructose	54.4	344	0.96	22
1.0 M	1.0 M Glucose	55.6	433	0.95	31
1.0 M	1.0 M Sucrose	56.3	444	1.0	32
1.0 M	1.0 M Trehalose	56.8	444	1.0	33
NaClO_4_					
*1.0 M*	*-*	*53.0*	*403*	*0.96*	*27*
1.0 M	1.0 M Fructose	52.6	326	0.97	20
*1.0 M*	*1.0 M Glucose*	*54.5*	*400*	*0.95*	*27*
*1.0 M*	*1.0 M Sucrose*	*55.4*	*420*	*0.96*	*30*
1.0 M	1.0 M Trehalose	58.2	392	0.98	28
GdmCl					
*1.0 M*	*-*	*53.9*	*383*	*0.94*	*25*
1.0 M	1.0 M Fructose	57.6	391	0.99	28
*1.0 M*	*1.0 M Glucose*	*57.4*	*394*	*0.95*	*28*
*1.0 M*	*1.0 M Sucrose*	*58.6*	*392*	*0.94*	*28*
1.0 M	1.0 M Trehalose	61.1	382	0.96	28
GdmSCN					
*0.5 M*	*-*	*47.9*	*320*	*0.94*	*17*
0.5 M	1.0 M Fructose	50.3	320	0.97	19
*0.5 M*	*1.0 M Glucose*	*50.0*	*324*	*0.93*	*19*
*0.5 M*	*1.0 M Sucrose*	*51.0*	*343*	*0.94*	*21*
0.5 M	1.0 M Trehalose	53.6	340	0.97	22

^a^ The errors on T_d_ and ΔH_d_^cal^ are ±0.2 °C and within 10%, respectively.

**Table 3:** Classic SPT estimates of the reversible work to create, in the reported aqueous solutions at 25 °C and 1 atm, cavities corresponding to the N-state (i.e., a sphere of 15 Å radius) and to the D-state (i.e., a prolate spherocylinder of 6 Å radius and 117 Å cylindrical length), respectively; values of ΔΔG_c_′ = ΔΔG_c_(co-solute) − ΔΔG_c_(water). All the numbers are in kJ mol^−1^ units; see text for further details.

	ΔGc(N)	ΔGc(D)	ΔΔGc	ΔΔGc′
**H2O**	1074	1878	804	-
1 M fructose	1125	1967	842	38
1 M glucose	1145	2001	856	52
1 M sucrose	1155	2020	865	61
1 M trehalose	1223	2138	915	111
**1 M urea**	1111	1942	831	27
1 M urea + 1 M fructose	1167	2040	873	69
1 M urea + 1 M glucose	1195	2088	893	89
1 M urea + 1 M sucrose	1207	2110	903	99
1 M urea + 1 M trehalose	1269	2218	949	145
**1 M TMU**	1079	1887	808	4
1 M TMU + 1 M fructose	1131	1978	847	43
1 M TMU + 1 M glucose	1168	2042	874	70
1 M TMU + 1 M sucrose	1165	2037	872	68
1 M TMU + 1 M trehalose	1237	2162	925	121
**1 M NaClO4**	1128	1972	844	40
1 M NaClO4 + 1 M fructose	1209	2114	905	101
1 M NaClO4 + 1 M glucose	1219	2130	911	107
1 M NaClO4 + 1 M sucrose	1230	2150	920	116
1 M NaClO4 + 1 M trehalose	1359	2373	1014	210
**1 M GdmCl**	1130	1976	846	42
1 M GdmCl + 1 M fructose	1187	2076	889	85
1 M GdmCl + 1 M glucose	1225	2141	916	112
1 M GdmCl + 1 M sucrose	1227	2145	918	114
1 M GdmCl + 1 M trehalose	1309	2288	979	175
**0.5 M GdmSCN**	1085	1897	812	8
0.5 M GdmSCN + 1 M fructose	1135	1985	850	46
0.5 M GdmSCN + 1 M glucose	1168	2041	873	69
0.5 M GdmSCN + 1 M sucrose	1188	2077	889	85
0.5 M GdmSCN + 1 M trehalose	1231	2151	920	116
